# The Digital Divide in Brazil and Barriers to Telehealth and Equal Digital Health Care: Analysis of Internet Access Using Publicly Available Data

**DOI:** 10.2196/42483

**Published:** 2023-07-21

**Authors:** Luis Filipe Nakayama, William Warr Binotti, Naira Link Woite, Chrystinne Oliveira Fernandes, Pia Gabrielle Alfonso, Leo Anthony Celi, Caio Vinicius Regatieri

**Affiliations:** 1 Department of Ophthalmology, São Paulo Federal University Sao Paulo Brazil; 2 Laboratory for Computational Physiology, Massachusetts Institute of Technology Cambridge, MA United States; 3 Department of Ophthalmology, New England Eye Center, Tufts Medical Center Boston, MA United States; 4 Sao Paulo University Sao Paulo Brazil; 5 Institute for Medical Engineering and Science, Massachusetts Institute of Technology Cambridge, MA United States; 6 Department of Informatics, Pontifical Catholic University of Rio de Janeiro Rio de Janeiro Brazil; 7 College of Medicine, University of the Philippines Manilla Philippines; 8 Department of Biostatistics, School of Public Health, Harvard TH Chan Boston, MA United States; 9 Department of Medicine, Beth Israel Deaconess Medical Center Boston, MA United States

**Keywords:** digital divide, digital health, health equity, internet access, medical care

## Abstract

**Background:**

The COVID-19 pandemic has increased the use of digital solutions in medical care, especially for patients in remote areas and those requiring regular medical care. However, internet access is essential for the implementation of digital health care. The digital divide is the unequal distribution of access to digital technology, and the first level digital divide encompasses structural barriers. Brazil, a country with economic inequality and uneven population distribution, faces challenges in achieving internet access for all.

**Objective:**

This study aims to provide a comprehensive overview of the first-level digital divide in Brazil, estimate the relationship between variables, and identify the challenges and opportunities for digital health care implementation.

**Methods:**

Data were retrieved from the Brazilian Institute of Geography and Statistics National Continuous House survey database, including demographic, health, and internet-related variables. Statistical analysis included 2-tailed *t* tests, chi-square, and multivariate logistic regression to assess associations between variables.

**Results:**

Our analysis included 279,382 interviews throughout Brazil. The sample included more houses from the northeast (n=99,553) and fewer houses from the central west (n=30,804). A total of 223,386 (80.13%) of the interviewed population used the internet, with urban areas having higher internet access (187,671/212,109, 88.48%) than rural areas (35,715/67,077, 53.24%). Among the internet users, those interviewed who lived in urban houses, were women, were younger, and had higher income had a statistically higher prevalence (*P*<.001). Cell phones were the most common device used to access the internet (141,874/143,836, 98.63%). Reasons for not using the internet included lack of interest, knowledge, availability, and cost, with regional variations. The prevalence of internet access also varied among races, with 84,747 of 98,968 (85.63%) White respondents having access, compared to 22,234 of 28,272 (78.64%) Black respondents, 113,518 of 148,191 (76.6%) multiracial respondents, and 2887 of 3755 (76.88%) other respondents. In the southeast, central west, and south regions, the numbers of people with internet access were 49,790 of 56,298 (88.44%), 27,209 of 30,782 (88.39%), and 27,035 of 31,226 (86.58%), respectively, and in the north and northeast, 45,038 of 61,404 (73.35%) and 74,314 of 99,476 (74.7%). The income of internet users was twice the income of internet nonusers. Among those with diabetes-related limitations in daily activities, 945 of 2377 (39.75%) did not have internet access, and among those with daily activity restrictions, 1381 of 3644 (37.89%) did not have access. In a multivariate logistic regression analysis, women (odds ratio [OR] 1.147, 95% CI 0.118-0.156; *P*<.001), urban households (OR 6.743, 95% CI 1.888-1.929; *P*<.001), and those earning more than the minimum wage (OR 2.087, 95% CI 0.716-0.756; *P*<.01) had a positive association with internet access.

**Conclusions:**

Brazil’s diverse regions have different demographic distributions, house characteristics, and internet access levels, requiring targeted measures to address the first-level digital divide in rural areas and reduce inequalities in digital health solutions. Older people, poor, and rural populations face the greatest challenges in the first level digital divide in Brazil, highlighting the need to tackle the digital divide in order to promote equitable access to digital health care.

## Introduction

The COVID-19 pandemic accelerated the adoption of technological solutions in health care to address the whole-city lockdowns, social distancing measures, and the interruption of ambulatory care elective surgeries [1,2]. In response, digital health care, which includes electronic health, mobile health, telemedicine, and telehealth, has emerged as a critical tool for promoting access to care, improving cost-effectiveness, and delivering better health outcomes [3-5].

Digital health care is capable of providing health care to patients living in remote and rural areas, patients with functional limitations, and patients who require regular medical care [6,7]. Technological advances, including remote monitoring, wearables, and artificial intelligence-assisted devices, have enabled the creation of a connected health care system that bridges individuals with their health care providers with real-time analysis of health care data, enabling the advancement of precision medicine and closer monitoring of chronic diseases [7,8].

However, the implementation of digital health care is not without its challenges. One of the most pressing is the digital divide, which refers to the unequal distribution of access to digital technology [5,9]. This divide is often divided into levels. The first level encompasses structural barriers, such as lack of internet connectivity, limited access to computer devices, software, and peripheral equipment, as well as a lack of motivation to use the internet [10,11]. The second level pertains to technical skills and the ability to effectively use digital technology [10,12]. These levels of the digital divide intersect and compound each other, leading to further inequities in access to digital technology and the opportunities it presents.

Despite increasing internet access and the growing popularity of mobile broadband, the first-level digital divide remains a significant challenge, particularly in low- and middle-income countries. The overall world internet access was 60% in 2020, with lower access in poorer countries [10,13].

Brazil, a middle-income country with a population of more than 214 million inhabitants, faces unique challenges in implementing technological solutions due to economic inequality and uneven population distribution. Although internet access was first implemented in Brazil in 1988, it has spread nonuniformly across the country, with the first-level digital divide affecting a significant portion of the population [14].

The Brazilian Federal Medicine Council has recently regulated telemedicine practice in Brazil, highlighting the potential for digital health care to address some of the challenges of the current health care system. However, internet access, which is a critical component of digital health care, remains uneven throughout the country, and addressing the first-level digital divide will be essential for the successful implementation of digital health care [15].

This study aims to provide a comprehensive overview of the first-level digital divide in Brazil, estimate the relationship between variables, and identify the challenges and opportunities for digital health care implementation.

## Methods

### Data Source

In this study, we analyzed demographic data retrieved from the Brazilian Institute of Geography and Statistics (IBGE) database according to the latest available data. The IBGE is a Brazilian government agency that provides data and information about the country for public consultation and research [16].

We included data from the most recent IBGE National Continuous Household survey database, covering the period of January 1, 2019, to December 31, 2019, which was prepandemic. We did not apply any exclusion criteria, and only selected variables were analyzed.

### Ethics Approval

This study was approved by the Sao Paulo Federal University ethics committee (33842220.7.0000.5505). This study used only fully anonymized publicly available data and therefore was exempt from informed consent by the institutional review board. The IBGE, a Federal Government Institution, collects, deidentifies, and shares the data.

### Data Collection

The data were collected quarterly using a direct population and a national household sample survey, which was conducted in person.

The house survey consisted of probabilistic samples of house units. The survey was stratified based on geographic strata and demographic distribution (age and sex). The variance and variance coefficients were calculated using linearization through the Taylor Series method. The survey included probabilistic samples of households, and the IBGE used probability sampling to extrapolate the findings to the whole country [17].

Six major interview themes were identified: general population characteristics, education level, work, household expenses, housing features, and access to the internet [18]. The responses to all questions were self-reported.

### Variables

The variables used in this study were extracted from the IBGE database using the *PNSIBGE* R package. They included demographic information, internet access characteristics, and health characteristics (Textbox 1). The IBGE divides Brazil into 5 macro-regions: the north (with 7 states), the northeast (with 9 states), the central west (with 3 states and the federal district), the southeast (with 4 states), and the south (with 3 states) [19].

**Variables extracted from the Brazilian Institute of Geography and Statistics database.**

**Demographics**

- Sex
- Race
- Macro-region
- Age (10-18 years, 19-40 years, 41-60 years, and more than 60 years)
- House type (urban and rural)
- Per capita year income (in US $; less than or higher than the minimum wage annual salary)

**Internet users**

- Internet access

**Health care**

- Limitations in daily activities
- Diabetes-related limitations in daily activities

The continuous age variable was grouped into 4 categories: 10-18 years, 19-40 years, 41-60 years, and more than 60 years, as well as a binary variable for older versus younger.

The income variable was converted to US dollars (BRL 1=US $0.184) and was grouped into 2 categories: less than the minimum wage (US $2203 per year) or higher than the minimum wage. Diabetes-related limitations in daily activities were classified as no, few, moderate, intense, and highly intense, according to reported core activity limitations. All levels of diabetic restriction and daily activity limitations were grouped for the analysis.

We considered the first-level digital divide and internet access in any device, and before statistical analysis, we assessed missing values for each variable and decided to delete or replace them based on the number of missing values.

In the analysis, we considered internet access as the outcome variable and region, house type, sex, race, and income as exposures.

### Statistical Approach

We performed descriptive statistical analyses to compare groups. We used *t* tests for continuous variables and chi-square tests to examine the association between dependent and independent variables. To assess the predictors’ weights, we built a multivariate logistic regression model with the included house type, age group, older vs younger age, region, race, and sex as independent variables and internet use as the outcome. We conducted a logistic regression analysis for each factor, followed by a multicollinearity analysis using the stepwise method, which considered every independent factor to determine the variable’s relationship. We used a 2-sided test for all hypothesis testing with a statistical significance level of α=.05.

Data were retrieved from the IBGE database using the R (version 4.2.2; R Foundation for Statistical Computing) *PNSIBGE* package, and analyses were performed using SPSS (version 27.0; IBM Inc) and Python (version 3.9) packages stats, *statsmodel*, and *researchpy*.

## Results

### Overview

The 2019 IBGE house survey conducted 279,382 interviews in 27 Brazilian states, representing around 4% of the country’s population, of which 838,146 people were included in the study; the north, northeast, central west, southeast, and south regions respectively accounted for 61,404 of 279,186 (21%), 99,476 of 279,186 (35.63%), 30,782 of 279,186 (11.02%), 56,298 of 279,186 (20.16%), and 31,226 of 279,186 (11.12%) of the interviewed Brazilian households.

### Missing Values

In the 2019 IBGE house survey data, there were 24 of 279,186 (0.008%) missing race variables and 172 of 279,186 (0.06%) missing annual income variables. For the variables age, internet access, house type, sex, and state, there were no missing values.

The mechanism behind missing data is not reported in the IBGE house survey data set. For the race and annual income variables (<5%), the rows with missing variables were deleted.

### Demographics

In 2019, Brazil had an estimated population of 209.5 million (48.2% male). For race, 148,191 of 279,186 (53.07%) of the interviewed Brazilians identified as multiracial, 98,968 of 279,186 (35.45%) as White, 28,272 of 279,186 (10.13%) as Black, and 3755 of 279,186 (1.34%) as other races. The age analysis showed that 71,516 of 279,186 (25.61%) people in the population were younger than 18 years, 91,806 of 279,186 (32.88)% were aged between 19 and 40 years, 72,335 of 279,186 (25.91%) were aged between 41 and 60 years, and 43,529 of 279,186 (15.59)% were older than 60 years. The majority of the population in all regions fell in the 19-40 year age group and lived in urban areas.

**Figure 1:**
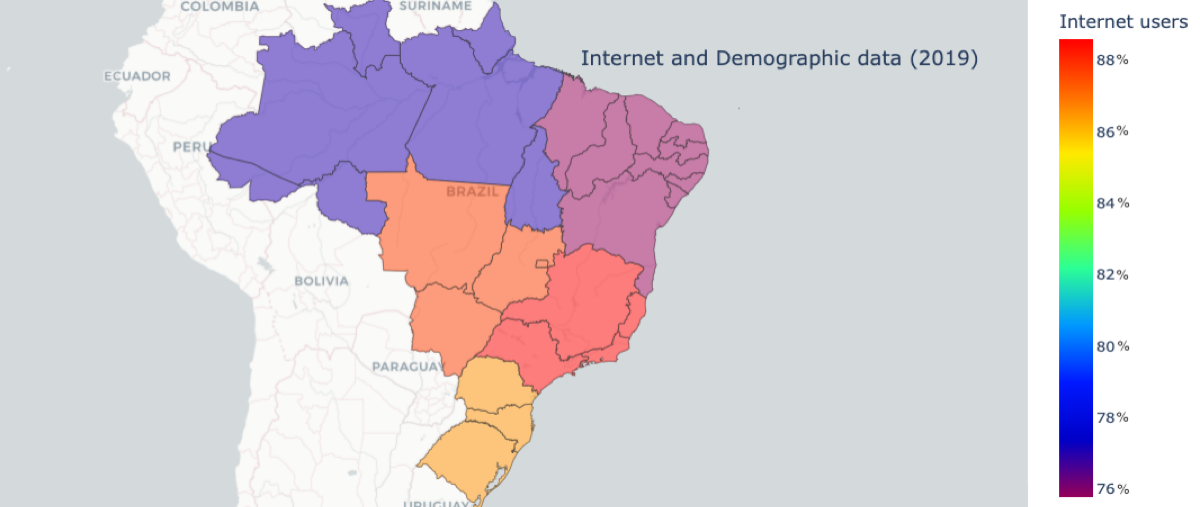
Map of Brazil with percentages of internet users (adapted from ©OpenStreetMap, licensed under the Open Data Commons Open Database License [20]).

Regional analysis showed that women outnumbered men in all regions. White respondents were more common in the southeast and south region, while multiracial respondents were more common in the north, northeast, and central west region. Urban areas were more common in all Brazilian macro-regions, although the percentage varied from 67,427 of 99,476 (67.78%) in the northeast region to 46,618 of 56,298 (86.36%) in the southeast region. Most of these households had access to the internet, ranging from 74,314 of 99,476 (74.7%) of homes in the northeast region to 49,790 of 56,298 (88.44%) of homes in the southeast region (Figure 1).

### Internet Users

Overall, 223,386 of 279,186 (80.13%) of the population in Brazil uses the internet, with 187,671 of 212,109 (88.48%) of urban and 35,715 of 67,077 (53.24%) of rural households having internet access, with a statistical difference (*P*<.001) between house types in accessing the internet. Urban internet users are more prevalent in the southeast region (44,363/48,618, 95.17%) and less prevalent in the northeast region (57,449/67,427, 85.2%).

In the sex comparison, the percentage of internet users was higher among women (117,379/144,790, 81.07%), with a statistical difference between the sexes (*P*<.001) in accessing the internet (Table 1).

**Table 1:** Characteristics and comparison of the total population, internet users, and non–internet users (n=279,382). Percentages for internet users and non–internet users are versus the total value in each row.

Variables and categories			Total		Internet users		Non–internet users		Chi-square (*df*)		*P* value
Age (years), mean (SD)			35.26 (21.67)		33.91 (20.59)		40.67 (24.83)		N/A^a^		N/A
**Older age (60+ years), n (%)**											
	Older age	43,529 (15.6)		28,007 (64.34)		15,522 (35.66)		7920.55 (1)		<.001	
**Sex, n (%)**											
	Male	144,790 (48.14)		106,007 (78.88)		28,389 (21.12)		209.38 (1)		<.001	
	Female	134,396 (51.86)		117,379 (81.07)		27,411 (18.93)		—^b^		—	
**House type, n (%) **											
	Urban	212,109 (75.97)		187,671 (88.48)		24,438 (11.52)		39,559.87 (1)		<.001	
	Rural	67,077 (24.02)		35,715 (53.24)		31,362 (46.75)		—		—	
**Race, n (%) **											
	White	98,968 (35.45)		84,747 (85.63)		14,221 (14.36)		3304.20 (4)		—	
	Black	28,272 (10.13)		22,234 (78.64)		6308 (21.36)				<.001	
	Multiracial	148,191 (53.07)		113,518 (76.6)		34,673 (23.4)				<.001	
	Other	3755 (1.34)		2887 (76.88)		868 (23.11)				<.001	
Yearly income (US $), mean (SD)			2901 (5095.36)		3248.60 (5557.02)		1510.90 (1964.24)		N/A		N/A
**Income groups, n (%)**											
	More than minimum wage	114,242 (40.92)		98,866 (86.54)		15,376 (13.46)		—		<.001	
	Less than minimum wage	164,944 (59.08)		124,520 (75.49)		40,424 (24.51)		—		—	
**Macro-regions, n (%)**											
	North	61,404 (21.99)		45,038 (73.35)		16,366 (26.65)		8147.45 (4)		<.001	
	Northeast	99,476 (35.63)		74,314 (74.7)		25,162 (25.3)				<.001	
	Central west	30,782 (11.02)		27,209 (88.39)		3573 (11.61)				.83	
	Southeast	56,298 (20.16)		49,790 (88.44)		6508 (11.56)				—	
	South	31,226 (11.12)		27,035 (86.58)		4191 (13.42)				<.001	
**Health variables, n (%)**											
	Diabetic restriction	2377 (0.85)		1432 (60.24)		945 (39.75)		59.81 (1)		<.001	
	Daily activity restriction	3644 (1.3)		2263 (62.1)		1381 (37.89)		25.90 (1)		<.001	

^a^N/A: not applicable.

^b^Not available.

The mean age of internet users was 33.91 years, while non–internet users were, on average, 40.67 years old. The older population made up 43,529 of the 279,186 (15.6%) total interviewed population, with a statistical difference (*P*<.001) between older and younger people in accessing the internet.

Internet users in Brazil had a per capita income of US $3248.60 per year, which is twice as high as non–internet users. Of the total interviewed population, 114,242 of 279,186 (59.08%) had less than the minimum recommended annual wage, with a statistical difference between income groups (*P*<.001) in accessing the internet.

For race, White respondents had a higher percentage of internet users (84,747/223,386, 85.63%), and multiracial respondents had a lower percentage (113,518/223,386, 76.6%), with a statistical difference (*P*<.001) between races in accessing the internet.

In the regional analysis, the southeast region had the higher percentage of internet users (49,790/56,298, 88.44%), and the north region had the lowest percentage (45,038/61,404, 73.34%). There was a statistical difference in internet access in the north, northeast, and south regions compared to the southeast.

The most common device used to access the internet in Brazil was the cellphone (141,874/143,836, 99.5%). Computers were more common in the southeast and south regions. Overall, the most common type of internet connection was mobile broadband or a mobile network (118,252/143,836, 82.21%), except in the northeast region, where cable broadband was more common.

The most common reasons reported by interviewees for not accessing the internet were lack of interest (11,574/36,979, 31.3%), lack of knowledge (15,666/36,979, 42.36%), and cost of internet access (7108/36,979, 19.22%). In regional analysis, the most reported reason for not accessing the internet varied. In the north region, the most common reason was the lack of internet availability. In the northeast, it was the cost of internet access. In the southeast, south, and central west regions, the most reported reason was a lack of interest.

### Health Parameters

Out of the 7101 houses included in the study, 2377 reported diabetes-related restrictions, while 4724 reported no such restrictions. In terms of daily activity restrictions, 3644 reported restrictions, while 5603 reported no restrictions.

Among those with diabetes-related limitations in daily activities, 945 of 2377 (39.75%) reported not having internet access, with a statistical difference between groups (*P*<.001) in accessing the internet. Similarly, among those with daily activity restrictions, 1381 of 3644 (37.89%) reported not having internet access, with a statistical difference between groups (*P*<.001) in accessing the internet.

### Logistic Regression

The logistic regression analysis showed that several factors were associated with internet access. In the univariate analysis, urban households were positively associated with internet access (odds ratio [OR] 6.743, 95% CI 1.888-1.929), while people aged older than 60 years had a negative association (OR 0.436, 95% CI –0.857 to –0.803). The 19-40 (OR 1.472, 95% CI 0.360-0.413) year and 40-59 (OR 1.061, 95% CI 0.033-0.086) year age groups were positively associated with internet access compared to the younger-than-18-years age group. The income group receiving more than the minimum wage (OR 2.087, 95% CI 0.716-0.756) was also positively associated with internet access. White respondents had a positive association (OR 1.618, 95% CI 0.448-0.515), while multiracial respondents (OR 0.889, 95% CI –0.148 to –0.087) and respondents of other races (OR 0.903, 95% CI –0.183 to –0.021) had negative associations compared to Black respondents. The north (OR 0.361, 95% CI –1.057 to –0.979), northeast (OR 0.338, 95% CI –0.985 to –0.909), and south (OR 0.847, 95% CI –0.214 to –0.118) regions had negative associations with internet access compared to the central west region (Table 2).

In the multivariate stepwise logistic regression, after adjusting for other factors, the 19-40 year age group, the south and southeast regions, and multiracial respondents and respondents of other races were no longer statistically significant (Table 2).

**Table 2:** Univariate and multivariate logistic regression of variables.

	Variable			Odds ratio (95% CI)		*P* value
**Univariate logistic regression**						
	**Sex**					
		Female	1.147 (0.118 to 0.156)		<.001	
	**House type**					
		Urban	6.743 (1.888 to 1.929)		<.001	
	**Age groups (years)**					
		19-40	1.472 (0.360 to 0.413)		<.001	
		40-59	1.061 (0.033 to 0.086)		<.001	
		≥60	0.436 (–0.857 to –0.803)		<.001	
	**Income**					
		More than minimum wage	2.087 (0.716 to 0.756)		<.001	
	**Region**					
		North	0.361 (–1.057 to –0.979)		<.001	
		Northeast	0.338 (–0.985 to –0.909)		<.001	
		South	0.847 (–0.214 to –0.118)		<.001	
		Southeast	1.005 (–0.039 to 0.048)		.83	
	**Race**					
		White	1.618 (0.448 to 0.515)		<.001	
		Multiracial	0.889 (–0.148 to –0.087)		<.001	
		Other	0.903 (–0.183 to –0.021)		.014	
**Stepwise multivariate logistic regression**						
	**Sex **					
		Female	1.100 (0.019 to 0.171)		.01	
	**House type**					
		Urban	3.393 (1.037 to 1.406)		<.001	
	**Age groups (years)**					
		19-40	1.016 (–0.202 to 0.233)		.89	
		41-60	0.470 (–0.966 to –0.543)		<.001	
		≥60	0.265 (–1.599 to –1.061)		<.001	
	**Income**					
		More than minimum wage	1.955 (0.290 to 1.051)		<.001	
	**Region**					
		North	0.174 (–1.982 to –1.514)		<.001	
		Northeast	0.365 (–1.231 to –0.786)		<.001	
		South	1.303 (–0.067 to 0.596)		.12	
		Southeast	1.036 (–0.227 to 0.297)		.79	
	**Race**					
		White	1.323 (0.099 to 0.460)		<.001	
		Multiracial	1.136 (–0.037 to 0.293)		.13	
		Other	1.181 (–0.288 to 0.621)		.47	

## Discussion

### Overview

The first-level digital divide remains a problem in Brazil, with our findings revealing that households in rural areas, low-income individuals, and older people encounter substantial barriers to internet access. These groups are disproportionately impacted by the digital divide, highlighting the urgent need for targeted efforts to bridge this gap and ensure equitable access to technology and information. Digital health care has the potential to increase democratization and cost-effectiveness and improve patient care [5,21]. However, internet access remains a challenge in Brazil, with uneven access throughout the country [14].

### Principal Findings

This study shows that, although most of the population has access to the internet, there is still an unequal distribution of internet access, with access mainly concentrated in urban areas. Almost half of the rural population lacks internet access, and per capita income for internet users is more than twice that of non–internet users. There is not a straightforward solution and distinct policies are needed to address these differences.

Older adults are particularly vulnerable to the digital divide, which is compounded in Brazil, where 64% of the older population has internet access [4,9,22].

Similar to previous studies, in Brazil, mobile devices and mobile broadband were the most used type of device and connection, respectively, which highlights the importance of mobile-optimized digital care platforms and mobile health care devices in Brazil [23].

While the economic barrier is a challenge for non–internet users, the lack of internet coverage is a problem, mainly in the north region, which covers the Amazon area. However, new internet satellite technologies make it possible to deliver broadband coverage even in rural and remote areas, such as the Amazon forest.

Patients with diabetic-related restrictions and patients with limitations in daily activities have a lower percentage of internet users than the Brazilian average. These patients would largely benefit from accessing health care services remotely, yet nearly 40% of these patients do not have internet access at home.

The statistical analysis reveals that Brazilians living in rural areas and older people have less chance of accessing the internet. In comparison to the central west region, the north and northeast regions have less chance of accessing the internet, and White people have more chance of accessing the internet compared to Black people.

The analysis shows that the rural house type and older people have the highest odds ratio, indicating that targeted measures are needed for these specific population groups to reduce the digital divide in Brazil.

Brazil is the fifth largest nation in the world, with extreme socioeconomic inequality and health disparities disproportionately affecting certain subpopulations due to uneven population distribution. Regarding demographic characteristics, the north, northeast, and central west regions have a higher proportion of multiracial people, while the south and southeast regions have a higher proportion of White people.

### Strengths and Limitations

This study has some limitations. First, the data used is restricted to the year 2019, which is the most recently available information in the IBGE database and is before the COVID-19 pandemic, with future analysis needed to evaluate the pandemic impact. Second, the data were sourced from probability sampling using the national household sample survey, which may not be a representative sample of the target population, limiting generalizability. Third, the data collected were self-reported; therefore, there is no guarantee that the information provided is accurate. Fourth, the IBGE race distribution data is distinct from the National Institutes of Health race classification, which does not account for ambiguity in those who do not fit clearly into 1 category [24]. Last, the data come from a single federal government institution source that performs census and household surveys.

Despite the limitations, the findings remain important and relevant. A lack of access to the internet compounded by uneven population distribution and economic inequality perpetuates the digital divide and marginalizes certain populations who are likely to benefit from digital health care solutions.

### Future Directions

The World Health Organization, in its 2020-2025 Global Strategy on Digital Health, published guidelines to provide appropriate and sustainable adoption of digital health technologies to achieve equitable population health improvement and to underscore the urgent need to address barriers to the implementation of digital health technologies [25].

This study’s analysis of demographic data is useful for understanding the population and tailoring public strategies. Internet access is a complex, multifaceted problem. Future studies are needed to understand how to improve digital health care access and evaluate internet access variation during the period and prospective analyses.

### Conclusion

In conclusion, Brazil is a large country characterized by economic inequality and uneven population distribution. Each sociodemographic category and geographic area has distinct needs to improve access to digital health technology and to enable fair and equitable digital health implementation.

Although technological advances offer promising opportunities to bridge gaps in care, internet access is not uniformly spread throughout the country, posing a significant challenge to digital health care. To promote equal access and not exacerbate existing health disparities, addressing the first- and second-level digital divide is crucial. Our findings reveal that rural households, low-income, people and older people face significant obstacles to accessing the internet. Patients with diabetes restrictions and limitations in daily activities have less internet access than the general Brazilian population. Thus, targeted measures are necessary to reduce the first-level digital divide in the country and ensure that all patients benefit from digital health care solutions.

## Abbreviations

IBGE: Brazilian Institute of Geography and Statistics
OR: odds ratio

## Data Availability

All data that support this study’s findings are publicly available at the Brazilian Institute of Geography and Statistics [16]. All the study data, codes, and analysis are available online [26].
